# Intraoperative CT-assisted sacroiliac screws fixation for the treatment of posterior pelvic ring injury: a comparative study with conventional intraoperative imaging

**DOI:** 10.1038/s41598-022-22706-y

**Published:** 2022-10-22

**Authors:** Zhanyu Yang, Bin Sheng, Delong Liu, Xintong Chen, Rui Guan, Yiwei Wang, Chao Liu, Rui Xiao

**Affiliations:** 1 Department of Orthopedics, Hunan Provincial People’s Hospital (the First Affiliated Hospital of Hunan Normal University), No. 61 Jiefang West Road, Changsha, Hunan China; 2 Hunan Emergency Center, No. 90 Pingchuan Road, Changsha, Hunan China

**Keywords:** Bone, Trauma

## Abstract

Pelvic injuries refer to the disruption of the inherent structural and mechanical integrity of the pelvic ring. Sacroiliac screw fixation technique is often applied for the treatment of posterior pelvic ring injury, which is prone to the iatrogenic injury. This study will compare the intraoperative and postoperative variables of patients underwent sacroiliac screw fixation with intraoperative CT and conventional imaging to evaluate the effect. Thirty-two patients with posterior pelvic ring injury treated by sacroiliac screw fixation from January 2019 to December 2020 were enrolled in this retrospective study. All patients were divided into two groups according to the different assistance of intraoperative imaging. Subsequently, the correlative data were compared and analysed statistically. Twelves cases were assigned to intraoperative CT group, and the remaining twenty cases were assigned to conventional group. There was no significant difference in duration of surgery, fracture healing time, time to ambulation, complications, and Matta radiological score. There was statistically significant difference (p < 0.05) in sacroiliac screws insertion time, length of incision, intraoperative blood loss, quality of screws position, and adjusted Majeed functional score, especially in the sexual intercourse part. With the assistance of intraoperative CT, a less misposition and functional impairment could be achieved, and a more satisfactory curative effect could be developed. Sacroiliac screws fixation with intraoperative CT is a more accurate and ideal method to treat posterior pelvic ring injuries.

## Introduction

Pelvic injuries refer to the disruption of the inherent structural and mechanical integrity of the pelvic ring, accounting for 3% of all skeletal fractures^1,2^. The pelvic ring is composed of the sacrum, coccyx, and the two innominate bones, each including the pubis, ischium, and ilium. The innominate bones fuse to form the acetabulum and join anteriorly to form the pubic symphysis^3^. Despite the lack of bony synostosis, the pelvic ring is highly stable due to the presence of numerous strong and extensive attachments of muscles, tendons, and ligaments^4^. Therefore, the pelvic injuries raise more attention since it is most occurred in the setting of high energy and is frequently complicated with additional injuries elsewhere in the body^5^.

The posterior pelvic ring, a structure ranging from one sacroiliac joint to the other, confers more stability than the anterior counterparts^6^. Most of pelvic fractures do not require operative intervention due to the resulting stability of posterior pelvic ring, such as iliac wing fractures. Approximately 40% pelvic fractures are unstable and need immediate treatment, which are often involved in the disruption of the posterior ring^7^. It is reported that the injury of the posterior pelvic ring is significantly associated with the increased morbidity, mainly related to hemorrhagic shock caused by venous plexus injury^8^.

Over the past few decades, minimally invasive techniques (MIT) have gradually been applied to the injuries of posterior pelvic ring, such as percutaneous insertion of cannulated screws into the sacroiliac joint. Compared to traditional fixation, percutaneous screw fixation technique presents an excellent result, allowing a shorter duration of surgery, a smaller incision, less soft tissue damage and less bleeding^9^. There is a safe zone between the neuroforamen and the cortex of the sacrum, which is often wide enough to place sacroiliac screws. However, some patients accompanied with atypical anatomy have narrow corridors so that it is difficult to locate the safe zone during operation^10^. Therefore, sacroiliac screw fixation technique is prone to the iatrogenic complication of vascular and nerve injury^11^.

Various imaging techniques are used for intraoperative evaluation to avoid the malposition of internal plants. However, the conventional 2D imaging with mobile C-arm cannot obtain the view of the safe area directly^10^. As a result, although the frequency of fluoroscopy exposure is often increased, the incidence of complications cannot be significantly reduced. Intraoperative CT (iCT) can provide vivid and complete three-dimensional imaging and facilitate the location of safe area accurately. With the rail-bound CT scanner being gradually devoted into newly built operating room, iCT has become a reliable tool for evaluation, especially in spine surgery^12^.

The aim of this study was to compare the differences in intraoperative and postoperative variables between iCT-assisted sacroiliac screw fixation and conventional imaging-assisted one and explore the application for implant navigation.

## Material and methods

A retrospective pattern was conducted in this study. The cases were included according to the following inclusion criteria: (1) patients with posterior pelvic ring injuries hospitalized from January 2019 to December 2020; and (2) treatment with sacroiliac screws fixation. The exclusion criteria were as follows: patients with (1) pathologic or nonacute fractures; (2) incomplete radiographic evaluation; (3) unavailable clinical documentation; (4) excessive obesity (BMI > 32 kg/m^2^); and (5) extremely poor conditions or diseases. The same surgical team is responsible for preoperative preparation, standard posture, anesthesia, operation, intraoperative fluoroscopy, postoperative rehabilitation, and follow-up of all cases to reduce the interference of variables.

The Siemens SOMATOM Conficence RT Pro was used for intraoperative scanning in January 2019. The device, a standard 64-slice dual-energy photon large-aperture CT, with the Direct Density technique, greatly broadens the selection of scanning voltage, maintains the accuracy of dose measurement, and obtains more accurate image quality. In addition, the multi-functional hybrid operating room was equipped with a movable operating table and a mobile fluoroscopy C-arm, allowing various operations to be performed without changing positions.

**Figure 1:**
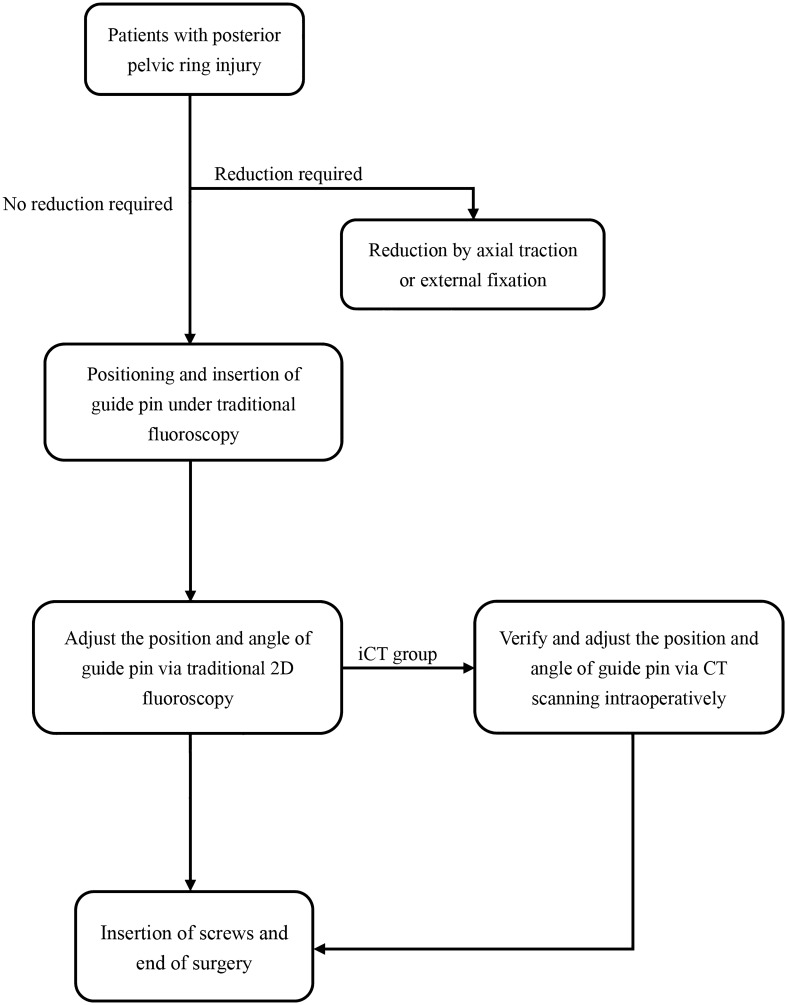
The operation workflow was presented.

**Figure 2:**
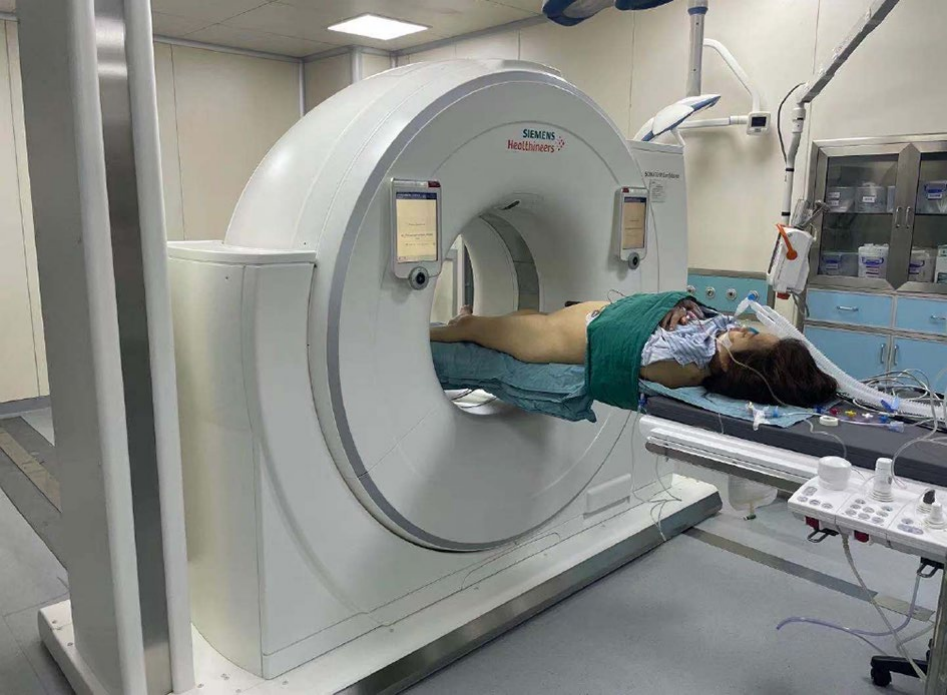
Standard posture of the patients with sacroiliac screw fixation navigated by intraoperative CT.

**Figure 3:**
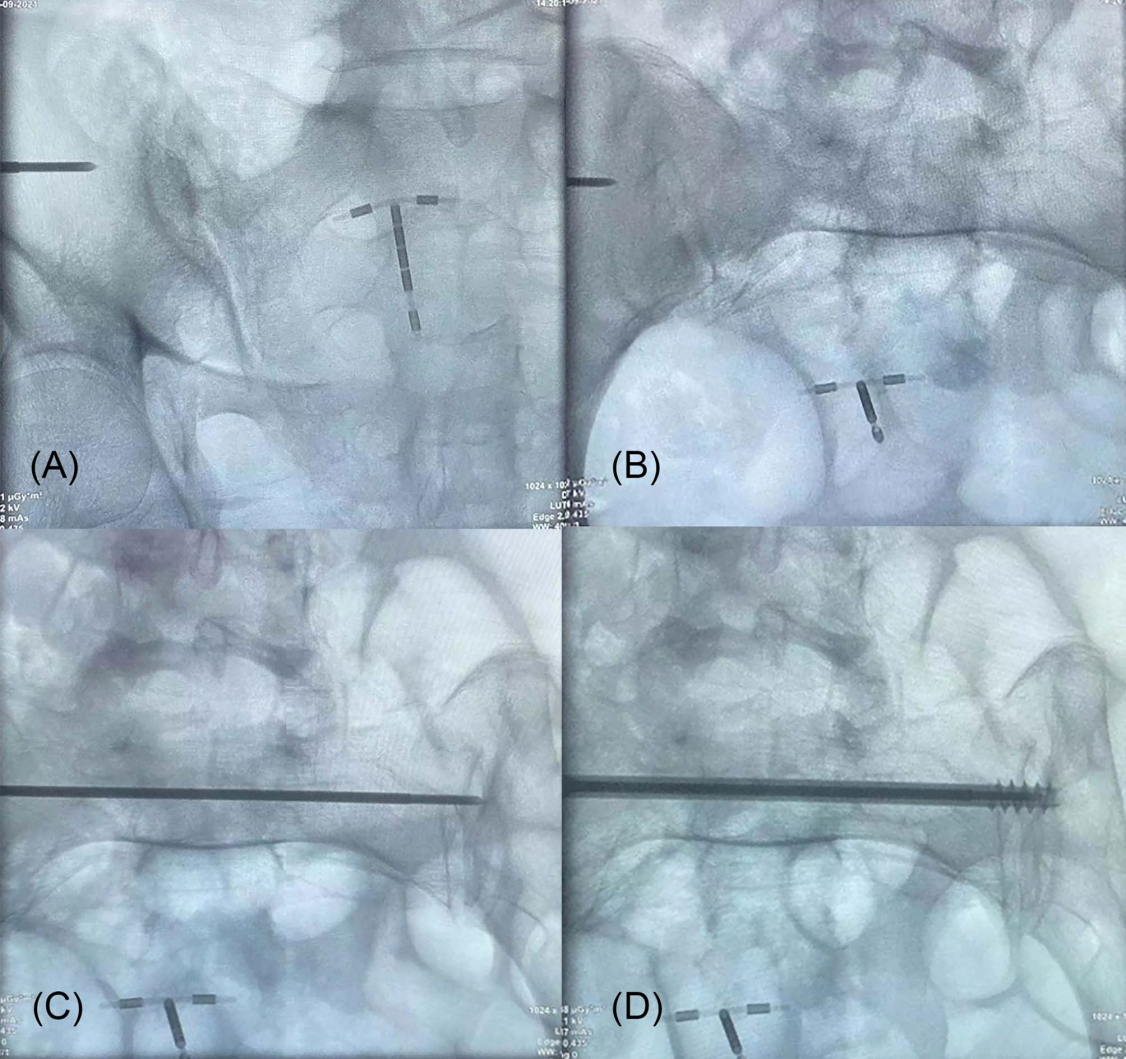
Radiographic imaging of the processes in sacroiliac screw fixation. (**A**) Pelvic outlet view. (**B**) Pelvic inlet view. (**C**) Placement of guided pin. (**D**) Placement of the sacroiliac screw in S1 across the bilateral sacroiliac joints.

**Figure 4:**
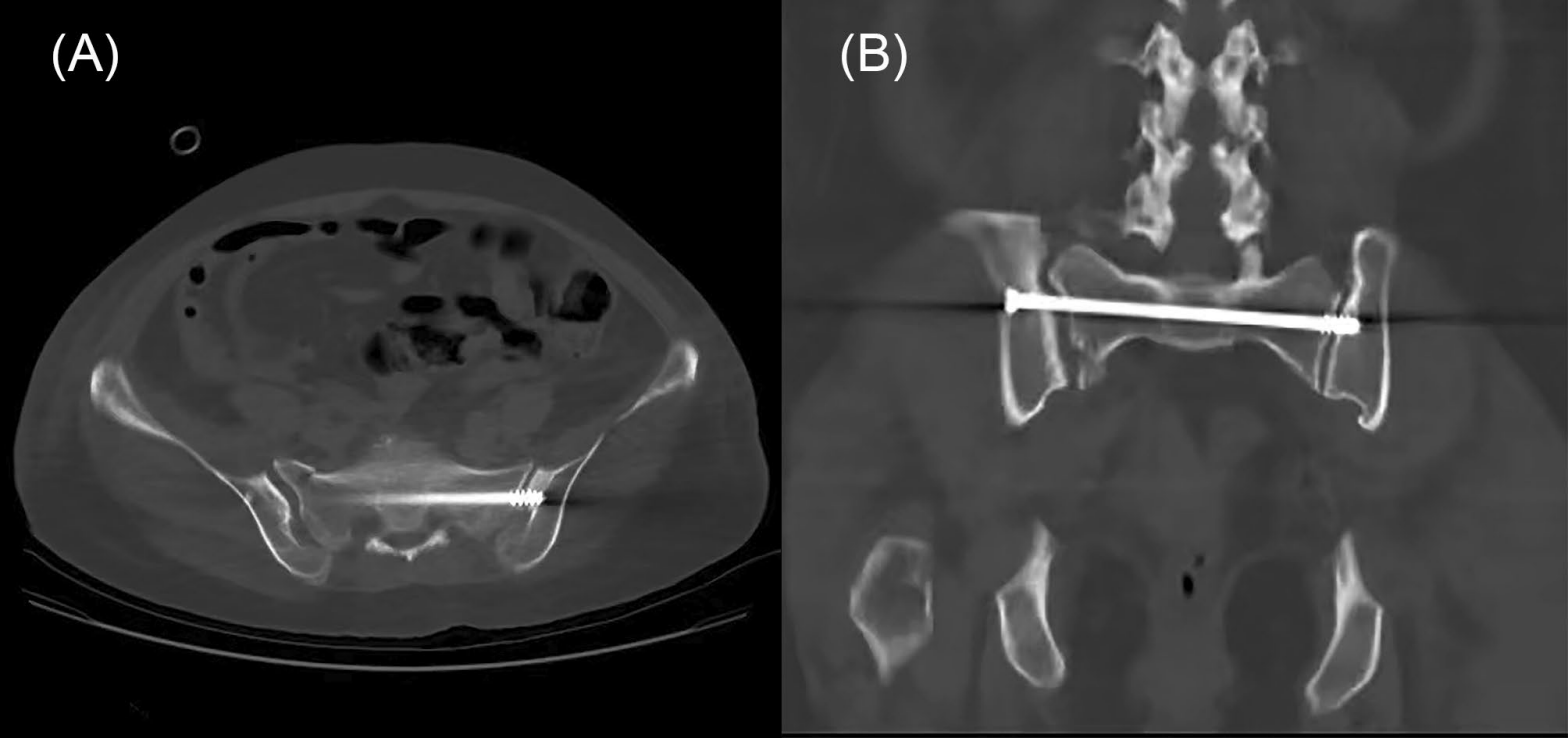
Imaging verification with rail-bound CT during operation. (**A**) Horizontal view. (**B**) Coronal view.

The included cases assessed and navigated with iCT and mobile C-arm were assigned to the iCT-assisted group, others only using mobile C-arm were assigned to the conventional group. As shown in Fig. 1, the operation workflow was presented. Standard posture of the patients with sacroiliac screw fixation navigated by intraoperative CT was shown in Fig. 2. For pelvic fractures that do not require reduction, sacroiliac screws can be directly inserted percutaneously. For pelvic fractures that need to be reduced, such as Tile C type, the height of hemipelvic ring in the injured side was restored by axial traction of the affected lower limb, and the rotational displacement was corrected by manipulating the iliac Schanz pins. After confirmation, the external fixation was used for temporary fixation to maintain reduction, and then sacroiliac screws were placed percutaneously. For unilateral sacroiliac joint injury, a single short screw is enough to obtain adequate stability. For sacral fractures, long screws are required. If the posterior pelvic ring is still unstable, double screws will be considered. When axial loading reaches 400 N to 800 N, no difference in posterior pelvic ring stability between the single- and double-screw fixation techniques. However, if combined with anterior pelvic ring injury, significant differences will emerge^13^. Stabilizing both S1 and S2, preferably with trans-sacral screws, could probably reduce the re-operation rate^14^. In addition, the screw can be placed into the S2 vertebral body on the premise that the pedicle diameter is large enough. Radiographic imaging of the processes in sacroiliac screw fixation was shown in Fig. 3 and then the placement of screws was verified with rail-bound CT as shown in Fig. 4. If the position of the guide pin is not good under iCT verification, the differences of the angle and distance were measured via computing technology and make accurate adjustment during operation promptly. All authors declare that all methods were carried out in accordance with relevant guidelines and regulations and all experimental protocols were approved by Hunan Provincial People's Hospital ethics committee. Written informed consent was obtained from all subjects or their legal guardians.

General information (age, gender, fracture classification, concurrent fractures of other parts, type of fixation and Injury Severity Score (ISS)) and the following variables were collected for analysis:

### Intraoperative variables

#### duration of surgery (min)

The total time of all procedures applied in surgery.

#### sacroiliac screws insertion time (min)

The time from pin navigation to sacroiliac screws insertion, excluding other operation steps.

#### Length of incision (cm)

Total length of surgical incisions associated with sacroiliac screws fixation, excluding the other surgical incision simultaneously.

#### Intraoperative blood loss (mL)

The total volume of bleeding during sacroiliac screws insertion time, calculated by weighing the blood-soaked gauze. The amount of blood loss (g) = the weight of gauze after wiping all blood loss − the weight of dry gauze (1 g = 1 ml).

### Postoperative clinical indicators

#### Fracture healing time(wks)

The standard of fracture healing is that the X-ray has showed that the fracture line was fuzzy and there was continuous callus passing through the fracture line.

#### Time to ambulation (mon)

Time to painless walking without any assistance.

#### Quality of screws position

The assessment criteria of screws position were consistent with Gras et al.^15^, as shown in Table 1. The excellence rate of screws placement was used for analysis.

**Table 1:** The assessment criteria of screws placement.

Classification	Description
Excellent	Completely within the cortex of the sacrum without any contact to the sacral foramen
Good	Partially contact the cortical bone without piercing
Poor	Penetrating the cortical bone

#### Complications (n)

The total number of complications occurred postoperatively, such as wound discharge and infection, implant failure, nonunion, revision and iatrogenic injury.

#### Postoperative scores

Majeed functional score and Matta radiological score are the two most used scoring systems for evaluating outcomes after pelvic surgeries^16^. The Matta score did not report the absolute score, and thus, the proportion of excellent cases was adopted for analysis.

The data were processed using SPSS 26.0 statistical software (IBM, Armonk, USA). The t-Test was used to compare the measurement data with normal distribution and homogeneity of variance between groups. The Chi-Square Test was performed for comparison between groups with the composition ratio. Significance was assumed when p < 0.05.

### Ethics approval and consent to participate

This clinical study is a retrospective study. It only collects the clinical data of patients, does not interfere with the treatment of patients, and will not bring risks to patients. The researchers will do their best to protect the information provided by patients from disclosing personal privacy.

## Results

44 potentially eligible cases were identified, and then 12 cases were excluded. 32 cases of posterior pelvic ring injury with sacroiliac screws fixation were enrolled in this study. Of 12 cases were assisted by intraoperative CT for navigation and reduction, and the remaining 20 cases were simply applied the traditional mobile C-arm for imaging during surgery. All 32 cases were followed up for 12–24 months (mean 16.53 months) and no case died or was lost to follow-up during the follow-up period. No patient had sacral nerve injury symptoms preoperatively. The detail characteristic of all cases is shown in Table 2. The average age of these patients was 48.31. There was no significant difference in age, gender, fracture classification, type of fixation, or Injury Severity Score (ISS) between two groups. The majority of the patients (91.69% in iCT-assisted group and 85.00% in conventional group) simultaneously suffered from other fractures on admission. The combined fractures include limb fractures, hip fractures, acetabular fractures and anterior pelvic ring injuries.

**Table 2:** The detail characteristic of all cases.

Index	iCT-assisted group (n = 12)	Conventional group (n = 20)	P value
Age	47.00 ± 15.27	49.10 ± 11.81	0.666
**Gender**			
Female	3	8	
Male	9	12	0.387
**Fracture classification**			
Type B	10	18	
Type C	2	2	0.581
**Type of fixation**			
One screw	8	16	
Two screws	4	4	0.399
Injury severity score (ISS)	12.42 ± 5.60	15.10 ± 8.92	0.358

A comparison of outcome parameters between iCT-assisted group and conventional group is shown in Table 3. For intraoperative variables, there was statistically significant difference (p < 0.05) in sacroiliac screws insertion time, length of incision and intraoperative blood loss between two groups. The average sacroiliac screws insertion time in iCT-assisted group was 63.33 min (SD: 23.01), which was significantly shorter than that in conventional group (p = 0.015). However, the average duration of surgery was 135.58 min (SD: 52.05) in iCT-assisted group and 137.60 min (SD: 38.62) in conventional group. There was no difference between two groups. The average length of incision was 1.29 cm (SD: 0.39) in iCT-assisted group and 1.92 cm (SD: 0.70) in conventional group. The average intraoperative blood loss was 59.03 ml (SD: 26.90) and 88.60 ml (SD: 44.54).

**Table 3:** The comparison of outcome parameters.

Index	iCT-assisted group (n = 12)	Conventional group (n = 20)	P value
Sacroiliac screws insertion time (min)	63.33 ± 23.01	86.85 ± 26.16	**0.015**
Duration of operation (min)	135.58 ± 52.05	137.60 ± 38.62	0.901
Length of incision (cm)	1.29 ± 0.39	1.92 ± 0.70	**0.009**
Intraoperative blood loss (ml)	59.03 ± 26.90	88.60 ± 44.54	**0.047**
Fracture healing time(wks)	26.00 ± 12.28	31.85 ± 11.60	0.187
Time to ambulation (mon)	1.07 ± 0.37	1.05 ± 0.27	0.848
Quality of screws position	12 (100.00%)	14 (70.00%)	**0.035**
Complications (n)	1	6	0.151
Majeed functional score	82.00 ± 11.61	74.90 ± 10.31	0.082
Adjusted Majeed functional score	86.78 ± 7.16	77.87 ± 10.86	**0.040**
Matta radiological score	7 (58.33%)	7 (35.00%)	0.198

Significant values are in bold.

However, for postoperative clinical indicators, there was no statistically significant difference in fracture healing time and time to ambulation. According to Gras et al. The excellence rate of screws position in iCT-assisted group was 100.00%, which was significantly higher than that of conventional group (70.00%). All cases showed no nonunion in fracture associated with posterior pelvic ring injuries. There was one case of wound discharge in both groups. Two cases in conventional group underwent revision surgery due to the misplacement of the screws in the sacral foramina. Three cases developed symptoms of sacral nerve injury in conventional group, including one case of perineal numbness and two cases of erectile dysfunction (Table 4).

**Table 4:** The postoperative complications.

Complications	iCT-assisted group (n = 12)	Conventional group (n = 20)
Nonunion	0	0
Wound discharge	1	1
Revision	0	2
Sacral nerve injury	0	3

The Majeed functional score one year after surgery in iCT-assisted group was 62–97 points (mean ± SD, 82.00 ± 11.61), with the result being excellent in 7 cases and good in 5, corresponding to an excellence rate of 58.33%. The Majeed functional score was 57–91 points (mean ± SD, 74.90 ± 10.31), with the result being excellent in 7 cases, good in 11, and fair in 2, corresponding to an excellence rate of 35.00%. There was no statistically significant difference in the Majeed functional score and the excellence rate. However, the adjusted Majeed functional score was obtained by eliminating the cases that had no work before injury. The adjusted score of iCT-assisted group was significantly higher than that of conventional group (p = 0.040). Especially in the sexual intercourse part, the conventional group (mean ± SD, 2.40 ± 0.94) was significantly lower than iCT-assisted group (mean ± SD, 3.17 ± 0.72) (p = 0.021). The proportion of patients with excellent rating according to the postoperative Matta radiological score was no statistically significant difference between two groups.

## Discussion

The conventional fixation methods are subjected to more blood loss, longer procedure time, and higher risk of infection, which hinders the recovery and may lead to secondary injury. The minimally invasive method, sacroiliac screws fixation, is gradually substitute for conventional methods in the treatment of posterior pelvic ring injuries^17^. However, the method is challenging, necessitates precise placement of implants to avoid serious complications^18^. Only a minimal deviation can cause an accurate penetration of the S1 foramina or the presacral cortex when fixed with the sacroiliac screws^19^. To reduce the occurrence of iatrogenic injury, many methods have recently been introduced to assist the insertion of sacroiliac screws. Using 3D-based fluoroscopy for the navigation of sacroiliac screws is not advantageous, compared with traditional 2D fluoroscopy^20^. Robot-assisted techniques, such as TiRobot and Artis Zeego, can effectively reduce the operation time and fluoroscopy times, and ensure the safety of the operation, helping the surgeon to quickly master sacroiliac screws surgery^21,22^. There are even attempts to remotely operate robots for surgery via 5G technology^23^. However, it is not applicable for the patients that require reduction during operation.

Intraoperative CT is gradually being integrated into the construction of a new operating room, allowing intraoperative scanning to assist the surgery^24^. This study aims to explore the advantages and disadvantages of intraoperative CT-assisted sacroiliac screw fixation in the treatment of posterior pelvic ring injuries compared with conventional imaging methods by collecting intraoperative and postoperative variables.

No significant difference in age, gender, fracture classification, type of fixation and ISS was identified. It suggested that the interference of the basic characteristics of different patients, different pelvic injuries, and other general conditions on the outcomes was weakened as much as possible. Most of the fracture types in the included cases were Tile B, and a few were Tile C, including not only simple posterior pelvic ring fractures, but also the sacroiliac fracture-dislocation. However, for complete sacroiliac joint dislocation, sacroiliac screws therapy was not appropriate.

In term of the sacroiliac screws insertion time, the average time in our study was about 86.85 min, which was longer than that of experienced orthopedic surgeon. This discrepancy may be attributed to the fact that our surgical team just implemented this technique not long ago and was not yet skilled. The duration of the MIT procedures was significantly correlated with the surgeon's experience^25^. Therefore, the same surgical team was selected to control the relevant confounding factors for the outcomes. Moreover, it also implied that with the assistance of iCT, the training cycle could be shortened, the safety could be ensured, and young surgeons could be escorted during operation.

As for the operative duration, if combined with other fractures that require concomitant treatment, additional time spent on surgery of other fractures was contained. These additional procedures included the fixation of limb fractures, hip fractures, acetabular fractures, and anterior ring injuries. Previous studies have shown that the application of a new assistive technique in the navigation of internal fixation often leads to a potential increase in the length of operation due to the additional preparation and execution^26,27^. However, there was no difference in the total time of operation between iCT-assisted group and conventional group. Moreover, the time of sacroiliac screws implantation can be significant reduced with the aid of iCT. On the one hand, the main reason for this difference is most likely that the manipulators are inexperienced in the early stage, resulting in redundant surgical steps during operation. On the other hand, this extra time can be compensated by reducing the time to repeatedly adjust the fluoroscopy for assessing the orientation of screws. Even the hidden gaining of averting unexpected anesthesia and surgery to correct misplacement of screws have not been considered. Of course, in view of the small sample size, it requires more cases, more practice and more frequent use to further study the existence of this difference.

Although percutaneous sacroiliac screws fixation usually requires a smaller incision than plate fixation, allowing a minimal blood loss^28^. The length of incision and the intraoperative blood loss in iCT-assisted group was still significantly lower than those in conventional group. This was consistent with Long et al.^29^. The frequency of intraoperative fluoroscopy and the possibility of prolonging the incision due to poor positioning during operation can be reduced via the auxiliary correction of computational implements. However, the clinical significance of a mean difference of less than 1 cm remains to be discussed, since there was no statistical difference in the incidence of wound-related complications between the two groups. The fracture healing rate of all cases in the two groups was 100%, and there was no significant difference in fracture healing time and time to ambulation between the two groups. It was as similar as expected. Intraoperative CT offers many advantages for surgical procedures; however, there was no difference in fundamental conditions. The variation of comminution and displacement and blood supply around the fracture are the crucial factors that determines the duration of fracture healing, which in turn adversely affects the time to start walking on the ground after surgery^29,30^.

Regarding postoperative complications, no loosening and failure of screws was found in all cases, which is consistent with several biomechanical studies^31–33^. Studies have shown that the probability of implant failure is different when only the posterior ring fixation is used in the case of instability of the posterior and/or anterior ring instability. In elderly patients, the failure rate of single-screw fixation was 12% when the posterior ring was injured alone and can be as high as 55% when the anterior and posterior ring were unstable simultaneously^13^. In our study, wound discharge was found to be a common complication, mostly related to the liquefaction of fat at the wound site. None of them eventually developed into a serious deep wound infection.

The quality of screws position in all cases was assessed according to Gras et al. The excellence rate of iCT-assisted group was significantly higher than that of conventional group. Two revision cases in the conventional group were due to symptomatic misplacement of screws detected by postoperative CT scanning, which was not detected by intraoperative fluoroscopy. Blame it on poor imaging. The sacral foramen and the anterior borders of S1 and S2 were indistinct on plain radiographs. Unclear identification of the markers allowed for screw misplacement. The position of the implants is extremely important and requires great precision. After all, some severe functional impairment may result from a displacement of as little as 1 cm^34,35^. Imaging on plain radiograph is inherently limited to the machine itself, body position, imaging angle, fracture type, and even bowel conditions. This is also the main reason for iatrogenic injury caused by sacroiliac screws under conventional fluoroscopy, and it is also the problem that this study aims to solve.

In sacroiliac screws fixation, lateral femoral and sacral nerve injuries and cortical penetration were slightly common^36,37^. Nerve-related injuries and symptoms occur in up to 8% of patients receiving sacroiliac screw fixation^38^. Surgeons must be more cautious to avert iatrogenic injury when using sacroiliac screw fixation for the treatment of posterior pelvic ring injuries, and additional fluoroscopy may facilitate navigating during operation^39–41^. Three cases with different degrees of nerve injury symptoms were found in the conventional group after surgery, while none in the iCT-assisted group.

Most notably, the incidence of complications in traditional group was 30%, although there was no significant difference between the two groups. This may be mainly because it refers to the total number of complications occurred postoperatively, rather than the number of patients with complications. Because of the misplacement of the screws complicated with the sacral nerve injury symptoms, two patients in the conventional group underwent revision surgery. Of course, this non-significant difference did not rule out the influence of factors such as surgical team and small sample size.

The average score of Majeed functional score in iCT-assisted group and conventional group was 82.00 points and 74.90 points, respectively. The excellence rate was 58.33% and 35.00%, respectively. However, there was no significant difference in the Majeed functional score and corresponding excellence rate between two groups. Surprisingly, in iCT-assisted group the adjusted Majeed scores calculated from excluding the confounding factor of score of non-working personnel before surgery have shown significantly more favorable results, compared to the conventional group. Especially in the sexual intercourse part, the conventional group was significantly lower than iCT-assisted group. An obviously increased risk of adjacent nerves and blood vessels would occur when the fracture separation was larger than 10 mm^42^. A more precise reduction after sacroiliac screw fixation can be achieved in contrast to that after open reduction^43^. It suggested that sacroiliac screws fixation with the assistance of iCT for posterior pelvic injury achieve a more satisfactory curative effect and especially reduce the adverse impact on postoperative sexual life. According to the Matta score, there is no significant difference in image of reduction and healing between the two group. However, iCT could provide a better image quality in the critical anatomical regions such as the pelvis, and the possibility of screw placement into the sacral foramen can be almost 100% avoided.

Perhaps another issue that needs more attention is radiation dosage. It is well known that radiation exposure in medicine is by no means benign. It can cause diseases and injuries, whether to patients or surgeons, accounting for approximately 0.6% to 3% of the cancer cases^44^. The radiation dosage of CT is usually much higher than that of traditional fluoroscopy. However, the radiation dosage of these two methods in practical application needs to be considered comprehensively. It is reported that the radiation exposure of minimally invasive techniques (MIT) in spine surgery is higher than that of open surgery, while the total radiation exposure of iCT-assisted MIT during the whole process of treatment is not significantly different from MIT with fluoroscopy^25^. This may be related to the times of intraoperative fluoroscopy, duration of use, and the rate of preoperative and postoperative CT. Most MIT procedures require more fluoroscopy and time, in contrast there are lower preoperative and postoperative CT scans with the assistance of iCT. Potential damage from radiation exposure can also be minimized by various methods, such as wearing lead clothing and other protective equipment on non-surgical sites^45^. Furthermore, the surgeons are closest to the radiation source during the conventional MIT procedures, whereas for intraoperative CT, they are not. Finally, the use of iCT-assisted navigation can completely reduce the cost-effectiveness of revision surgery for symptomatic screw misplacement. Taking all these factors into account, the benefits of iCT-assisted MIT far outweigh the disadvantages.

There are also several limitations in current study. Firstly, our study was retrospective and observational, with relatively small sample sizes, in nature, which was prone to random variations and uncontrolled bias. The different degrees of injuries, variable fracture types or classifications, and different anterior fixation methods would have possibly affected the outcomes. Matched pair analyses can better control the effects of irrelevant variables and should have been chosen. However, due to the inherent limitations of retrospective studies, confounding factors cannot be effectively controlled to obtain reasonable matched groups. Secondly, not all cases were followed up for 24 months. The research subjects will continue be followed up. If feasible, a multicenter study will be performed, and more cases will be included in the future. Thirdly, only one device can be relied on, and more data originated from other similar systems is required. Finally, there is a limiting factor in the implementation and development of such a intraoperative CT, which requires high investment, so that it is relatively rare in general hospitals. In view of the above problems, trials with larger samples and higher quality can be carried out in the future to better control confounding variables and improve the reliability of findings. Furthermore, separate radiation protection guidelines should be proposed and followed to better protect patients.

## Conclusions

To summarize, our findings demonstrates that a significant reduction of length of incision and blood loss with intraoperative assistance of CT could be obtained without significantly prolonging duration of operation. Less misposition and functional impairment could be achieved, and a more satisfactory curative effect could be developed, especially in the postoperative sexual life. Therefore, we consider that sacroiliac screws fixation with intraoperative CT assistance is a more accurate and ideal method to treat posterior pelvic ring injuries.

## Data Availability

The datasets used and analysed during the current study available from the corresponding author on reasonable request.
